# Hide and seek with SARS-CoV-2: spike receptor-binding domain-specific memory B cells still recognize Omicron variant

**DOI:** 10.1038/s41392-022-01192-8

**Published:** 2022-10-02

**Authors:** Ivan Odak, Reinhold Förster, Berislav Bošnjak

**Affiliations:** 1 Institute of Immunology, Hannover Medical School, 30625 Hannover, Germany; 2 Cluster of Excellence RESIST (EXC 2155), Hannover Medical School, 30625 Hannover, Germany; 3 German Centre for Infection Research (DZIF), Partner site Hannover, Hannover, Germany

**Keywords:** Infectious diseases, Adaptive immunity, Vaccines

Sokal et al. reported recently in Immunity that the SARS-CoV-2 Omicron variant evades recognition by 70% of anti-spike protein antibodies generated by memory B cells (MBCs) from mRNA vaccine-immunized individuals that were either or not also infected with SARS-CoV-2. Nevertheless, samples from all analyzed persons contained MBCs with neutralizing antibodies against Omicron Spike receptor-binding domain (RBD), which provide protection against this SARS-CoV-2 variant to some degree.^1^

**Fig. 1:**
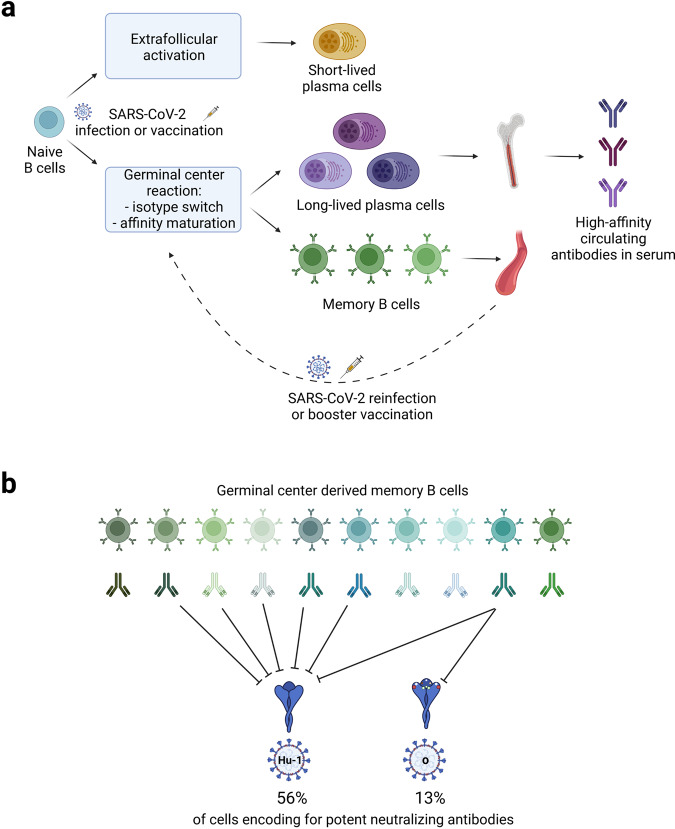
**a** Scheme of humoral immune responses to SARS-CoV-2 infection of vaccination. Initially, upon antigen encounter some naïve B cells mature into short-lived plasmablasts that migrate to lymph node medullary chords and produce a first wave of low-affinity antibodies. The full power of humoral immunity, however, relies on a germinal center reaction. A subset of B cells with the initially highest affinity for the invading pathogen moves to the center of a B cell follicle, where they begin to proliferate thereby forming a germinal center (GC). In the dark zone of the GC, where B cells rapidly proliferate, base-pair changes get introduced into the rearranged immunoglobulin variable regions of heavy- and light-chains genes in a process called somatic hypermutation. B cells encoding for antibodies with improved antigen binding affinity are then selected in the light zone of the GC with the help of T follicular helper cells that provide survival factors for the GC B cells and follicular dendritic cells that present non-degraded antigens. Several iterative rounds of B cell re-circulations between the light and dark GC zones ensure the generation of B cells with high-affinity antibodies. These cells will further differentiate into either long-lived plasma cells or memory B cells. Long-lived plasma cells migrate to bone marrow to secrete high-affinity antibodies in serum. In contrast, memory B cells remain circulating between blood, secondary lymphoid organs, and lymph and can reactivate after antigen re-counter and re-enter germinal center reaction. **b** Mutations accumulated in spike protein receptor-binding domain (RBD) of Omicron variant (O) evade a large fraction of high-affinity memory B cell derived antibodies induced by ancestral SARS-CoV-2 strain (Hu-1). Figure created with BioRender.com

Initiation of humoral immunity occurs in several steps (Fig. 1a). While low-affinity antibodies produced from short-lived plasmablasts provide protection at early stage post infection, production of high-specific antibodies requires B cells to go through a germinal center reaction. In the germinal centers, follicular dendritic cells and follicular helper T cells support B cells going through a somatic hypermutation, a process in which B cells encoding for antibodies with improved antigen binding affinity are selected (for details refer to Fig. 1a). Some of these cells differentiate to long-lived plasma cells (LLPCs) that move primarily to the bone marrow, and constitutively secrete antibodies into the blood to neutralize invading pathogens immediately upon reinfection. In addition, some B cells with antibodies of relatively low-affinity turn into circulating MBCs, ensuring that poly-reactivity against antigen is maintained to some degree. These cells not only provide a faster response to antigens/pathogens months or years after initial infection, MBCs are also crucial for retaining flexibility in terms of antigen binding as a countermeasure to pathogen immune evasion by antigenic drift. The complex GC reaction induced in response to infection is also the basis for the induction of potent humoral immunity after vaccination.

The word wide onset of COVID-19 vaccination campaigns allowed researchers to study the de novo formation of humoral immunity against SARS-CoV-2 and to compare the one found in vaccinated and non-vaccinated COVID-19 convalescent individuals. While many studies focused on the production of SARS-CoV-2-specific antibodies in serum, the group of Pascal Chappert and Matthieu Mahévas focused on virus-specific MBCs. Previously, they showed that SARS-CoV-2 infection induces not only high serum titers of anti-SARS-CoV-2 spike antibodies but also differentiation of circulating anti-spike MBCs.^2^ During 6 months following infection RBD-specific MBCs cells expanded and matured. They progressively accumulated mutations in variable regions but retained strong neutralizing potential against the ancestral SARS-CoV-2 strain (Hu-1).^3^ Importantly, mRNA vaccination boosted those cells, inducing a novel round of GC reaction and further mutation events in the variable regions of heavy chain (*V*_H_) domains.^3^ Almost all vaccine-induced MBCs containing >10 mutations within the *V*_H_ and produced mid- and high-affinity antibodies directed against the Hu-1 strain. Although some MBCs increased their antibody affinity, the overall diversity of the RBD-specific MBC repertoire was maintained and was similar to the repertoire of MBCs from virus-naive, vaccinated individuals. Depending on the donor, 5–60% of this broad MBC repertoire had high-affinity clones against Alpha, Beta, Gamma, and Delta variants of SARS-CoV-2.^3^ These data suggest that infection- or vaccination-induced MBCs may still provide neutralizing antibodies upon vaccination boost or reinfection with novel virus variants.

Viral escape, however, continued. Since the appearance of the latest SARS-CoV-2 Omicron variant numerous reports demonstrated that exposure to previous virus variants and/or vaccination provides only limited protection to infection and clinical disease manifestation. This is due to diminished ability of the antibodies to neutralize the heavily mutated Omicron RBD, especially because of N501, K417, and E484 mutations.^1^ However, Sokal *et al*. now provide evidence of incomplete immune evasion of Omicron to circulating MBCs.^1^ The authors shown that although approximately 70% of the analyzed MBC clones showed no or low affinity to Omicron RBD, approximately 30% of the antibodies encoded by the MBCs of convalescent and/or vaccinated individuals still retained high affinity. The reason for such a drastic drop in affinity lies in the fact that, compared to other variants, Omicron-specific mutations seem to affect a more diverse range of RBD-specific MBCs. Of note, potent neutralizers of other SARS-CoV-2 variants had the highest loss of affinity against Omicron RBD. Furthermore, the loss of neutralization potency was even more pronounced and only 13% of the high-affinity MBCs could still produce anti-Omicron-RBD neutralizing antibodies (Fig. 1b). Nevertheless, neutralizing antibodies were still present in all samples from all 11 tested donors, and 3 out of 4 donors that had severe COVID-19 and were boosted later with an mRNA vaccine harbored more than 20% of MBCs that produced potent neutralizing antibodies against Omicron RBD present in BA.1 variant.^1^ It would be important to evaluate further neutralization capacity of MBCs from a larger number of donors against even more heavily mutated Omicron variants such as BA.2.12.1, BA.4 and BA.5.

An important question that requires answer is whether these rare MBCs provide protection against Omicron (re)infection. A significant hurdle for their reactivation is immune imprinting (original antigenic sin). In this phenomenon when adaptive immunity to a particular antigen is boosted with a second, cross-reacting antigen, the ensuing recall responses react faster to the initial priming antigen, hampering the creation of de novo responses. Recent report supported the antigenic sin model in the context of SARS-CoV-2 vaccination. In that study, the authors boosted animals that had been vaccinated twice before with a Hu-1-based mRNA vaccine with either original vaccine or an Omicron-adapted version. When testing the sera collected sera 14 days after the booster injection for their ability to neutralize Omicron no differences could be observed.^4^ On the other hand, Pfizer and BioNTech have announced that Omicron-adapted vaccines still could induce higher immune responses against Omicron SARS-CoV-2 variant that the original COVID-19 mRNA vaccine.^5^ Hence, it would be important to collect samples from larger number of subjects after Omicron breakthrough infection or Omicron-specific booster vaccination to establish whether any of Omicron-specific MBCs can be expanded and prevent infection. In addition, it will be crucial to assess the effect of adapted SARS-CoV-2 vaccines on T cell responses, as well as those MBCs could be induced with protein-based or attenuated virus vaccines.
